# Deep Learning Classification for Diabetic Foot Thermograms †

**DOI:** 10.3390/s20061762

**Published:** 2020-03-22

**Authors:** Israel Cruz-Vega, Daniel Hernandez-Contreras, Hayde Peregrina-Barreto, Jose de Jesus Rangel-Magdaleno, Juan Manuel Ramirez-Cortes

**Affiliations:** 1 CONACYT Research Fellow—National Institute of Astrophysics, Optics, and Electronics, Santa Maria Tonantzintla, Puebla 72840, Mexico; 2 Department of Electronics, National Institute of Astrophysics, Optics, and Electronics, Santa Maria Tonantzintla, Puebla 72840, Mexico; 3 Department of Computational Science, National Institute of Astrophysics, Optics, and Electronics, Santa Maria Tonantzintla, Puebla 72840, Mexico

**Keywords:** thermography, artificial neural networks, support vector machine, deep learning, diabetes mellitus, diabetic foot

## Abstract

According to the World Health Organization (WHO), Diabetes Mellitus (DM) is one of the most prevalent diseases in the world. It is also associated with a high mortality index. Diabetic foot is one of its main complications, and it comprises the development of plantar ulcers that could result in an amputation. Several works report that thermography is useful to detect changes in the plantar temperature, which could give rise to a higher risk of ulceration. However, the plantar temperature distribution does not follow a particular pattern in diabetic patients, thereby making it difficult to measure the changes. Thus, there is an interest in improving the success of the analysis and classification methods that help to detect abnormal changes in the plantar temperature. All this leads to the use of computer-aided systems, such as those involved in artificial intelligence (AI), which operate with highly complex data structures. This paper compares machine learning-based techniques with Deep Learning (DL) structures. We tested common structures in the mode of transfer learning, including AlexNet and GoogleNet. Moreover, we designed a new DL-structure, which is trained from scratch and is able to reach higher values in terms of accuracy and other quality measures. The main goal of this work is to analyze the use of AI and DL for the classification of diabetic foot thermograms, highlighting their advantages and limitations. To the best of our knowledge, this is the first proposal of DL networks applied to the classification of diabetic foot thermograms. The experiments are conducted over thermograms of DM and control groups. After that, a multi-level classification is performed based on a previously reported thermal change index. The high accuracy obtained shows the usefulness of AI and DL as auxiliary tools to aid during the medical diagnosis.

## 1. Introduction

Diabetes Mellitus (DM) is one of the leading worldwide causes of death and degrading elements in the quality of life of those affected by the disease [1]. There are several complications associated with DM, including heart attacks, vision loss, kidney failure, and amputations of inferior limbs. Such difficulties not only affect people’s health, but they also have a significant impact on their personal and working life. Diabetic foot is one of the main complications. It has been reported that a loss of sensitivity in the diabetic foot, along with mechanical stress in the plantar region, may increase the risk of ulceration [2], which can lead to an amputation [3]. It is also known that an increase in temperature in the plantar region of diabetic patients is associated with a higher risk of ulceration [4]. Hence, the interest in monitoring the temperature frequently through different approaches has arisen [5,6].

Thermography is a technique that has been applied to the study of diabetic foot by analyzing the thermal changes that occur in the affected foot [5]. This technique presents two main advantages. Firstly, contact is not required, and, secondly, it is non-invasive. Several works concerning its use to study diabetic foot have been reported [7,8,9,10]. Two main approaches have been proposed for thermogram analysis. These involve identifying characteristic patterns and measuring thermal changes. On one hand, a control group has been demonstrated to show a particular spatial pattern, called butterfly pattern [11]. However, there is a wide variation of spatial patterns in the DM group [12,13]. On the other hand, it is possible to measure the thermal changes and to make an assessment of these with respect to a reference. Some works propose a contralateral comparison of temperature by assuming that one foot serves as a reference to the other [14,15,16,17]. However, the contralateral comparison is limited when one of the feet cannot serve as reference. For example, if both feet have changes in temperature, but neither one of them has the butterfly pattern, then one cannot be a reference of the other. If changes in both feet have similar spatial distributions, asymmetry will not be detected, even when there is a significant change in temperature. An alternative to this approach is to measure the changes by computing a representative value for each foot of the DM group and taking the butterfly pattern as reference. Therefore, the measurement depends on the temperature distribution, and not on a spatial pattern [18,19,20]. This kind of analysis can help to describe thermograms to improve their automatic classification and bring additional information to the medical expert.

Medical information systems are changing from traditional manual data analysis to expert computer-based analysis or Computer-Aided Diagnosis (CAD) for detection purposes and in support decision systems. The primary objective is to provide accurate diagnostic support tools for clinicians. Human visual capacity for providing a diagnosis based on clinical imaging is known to have limitations, and certain effects, such as optical illusions, could affect the diagnosis accuracy [21]. Other factors to take into account are errors due to negligence, fatigue, and sensory overload caused by the massive amount of information [22]. In addition, the shortage of specialists for the diagnostic task in many healthcare institutions is also a current problem.

Traditional techniques for automatic classification, such as Artificial Neural Networks (ANN) and Support Vector Machines (SVM), are commonly used for image classification and feature extraction [23,24,25]. Both techniques are black boxes but have relevant properties that include parallel multiprocessing and the use of data-transformation with kernels. They provide high-accuracy classification results, especially in the classification and processing of images. However, prior to these processes, they require previous steps, namely preprocessing and the subsequent extraction and selection of features. Currently, there is an interest in the use of Deep Learning Networks for several tasks in medical applications, such as feature extraction, semantic segmentation and classification, among others. The shift of attention from conventional paradigms in machine learning to DL is a result of the high accuracy achieved through its massive-learning-structures, which allow DL to obtain deeper traits of the data. However, some issues need to be addressed when DL is used; these include the dataset size, the appropriate labeling of the samples, the segmentation and selection of Regions Of Interest (ROIs), the use of pre-trained structures in the mode of transfer learning, or the design of a proper new learning-structure from scratch, among others.

Although it could be relatively easy to identify when a foot thermogram does not belong to the control group at a glance, it is difficult to say how much the distribution has changed since there is no spatial pattern that describes the changes in the diabetic foot. Hence, it is important to provide a measurement or description of the thermal change that does not depend on a spatial pattern. The following considerations must be taken into account: (i) the spatial distribution could change after some time, and, (ii) in certain cases, the thermal changes are not so evident. In fact, the distribution could be visually perceived as slightly different from the control. Thus, the visual assessment of the expert can be complemented with an analysis to identify and measure the thermal changes. Moreover, a trained net specialized in diabetic foot thermograms can help to automatize this task.

Regarding the temperature changes in the diabetic foot, we are interested in detecting abnormal temperature increases in thermograms of the plantar region for their classification. This provides relevant considerations for the comparison of the three techniques: Machine Learning (ML), Singular-Value Decompositon(SVD), and DL. For this, feature extraction will be performed by an automatic segmentation process derived from an evolutionary-computing algorithm and histogram-based thresholding method using fuzzy entropy measures. Moreover, the design of a new DL-structure called Diabetic Foot Thermogram Network (DFTNet) is proposed, presenting the best results in accuracy and other performance measures, such as specificity, Area Under the Curve (AUC), and precision, among others. In the case of classifying multiple levels of temperature, the neighboring classes are complicated to classify due to similarities of the thermograms; however, DFTNet outperforms the results of such complicated cases, even in the case of the complex structures of AlexNet and GoogLeNet. With these new proposals, we are extending our previous research on comparing the Multilayer Perceptron (MLP), SVM, and AlexNet for the classification of just DM patients against the healthy ones [26]. The new DFTNet and the classification of the additional five multiple levels of the disease represent this further improvement. In addition, the use of patches instead of complete images, allowed us to increase the database for at least five times, obtaining a sufficient size of the dataset to train the DFTNet from scratch. All of these results are backed up by the incorporation of new quality measures. The DFTNet is available as supplementary material of this work.

The content of the paper is described as follows. In Section 2, relevant findings in automatic segmentation and in the use of computational intelligence-based classifiers are addressed. The methodology in Section 3 describes the steps followed in this research and the description of the proposed DL-structure. Section 4 presents the final results and Section 5 describes a discussion. Finally, the conclusions and future directions are presented in Section 6.

## 2. Related Work

CAD systems are generally classified into offline and online methods [27]. Most of these CAD systems follow an offline process, which consists of preprocessing the images, extracting relevant features, analyzing statistical techniques, determining the highly significant features, and finally classifying them with either some manual techniques (like thresholding or the point-to-point mean difference technique) or by using computational intelligent methods (like Artificial Neural Networks or Fuzzy Logic). In the online system, the same distinct features are obtained, and the system tries to classify as the data comes in. The latter marks a recent use of applications, like those in smartphones, as an indicative tool, not as a diagnostic one [28,29].

In the next subsections, we mention some related works of the segmentation process and the classification based on computational intelligence. Both of these are of interest to this work.

### 2.1. Segmentation and Feature Extraction

Image segmentation and feature extraction are two crucial steps in computer-aided diagnosis systems. The selection of a suitable segmentation technique and the extraction of relevant features can increase the accuracy of the CAD systems. Scientific literature reports several thermogram segmentation processes to isolate the plantar region from the background. Kaabouch et al. [16] evaluated five groups of auto-thresholding techniques, namely histogram shaped-based methods, clustering-based methods, entropy-based methods, object attribute-based methods, and complex genetic algorithms, and concluded that the genetic algorithms based on the thresholding technique give the best results. In 2013, Liu et al. [30] implemented the methods described in [16], but the results obtained were not satisfactory in image since there was no clear intensity difference between the foot and the background. They implemented an Active Contours Without Edges (ACWE) method; however, the images without high contrast and visible parts of the ankles and legs still needed manual adjustment. Nandagopan et al. [31] presented a comparison between two segmentation methods applied in 10 thermograms of healthy feet and 10 of diabetic ones. The results showed that the edge detection technique was more reliable than the watershed method. In [32], the snake algorithm is used to isolate the plantar region from the thermograms. The snake algorithm serves, for both purposes, to separate the feet from the background and to separate the right foot from the left one into different segmented clusters according to their temperatures. The authors claim that this snake algorithm is more robust than conventional algorithms, such as Canny, Sobel, and Otsu-thresholding. Another work [33] presents a method based on a modified active contour model that includes prior shape information. The proposed method outperforms the classical snake method, as well as other segmentation methods, such as region growing, active contour approaches, level-based methods [34,35,36], and the graphic cut approach [37].

Image segmentation techniques can also play a crucial role to isolate the hottest region in plantar thermograms, which can be used to extract relevant features. A hot region in diabetic subjects can be a sign of tissue damage or inflammation. Etehadtavakol et al. [38] demonstrated the importance of extracting the hottest/coldest regions from thermographic images using lazy snapping. Lazy snapping is an interactive image algorithm that divides coarse and fine scale processing, accomplishing object condition and detailed adjustment effortlessly. In addition, lazy snapping contributes instant visual feedback, separating the divided contour from the accurate object boundary conveniently, regardless of the existence of low contrast edges. In [18], the authors used a histogram-shape based thresholding method to extract the hottest temperatures from the plantar region. After that, they formed a feature vector based on the components of the morphological pattern spectrum, including a position criterion. In [39], Gururajarao et al. used an active contour model for plantar segmentation and a thresholding method to extract the hottest region. They divided the plantar region into six areas and extracted features, such as correlation, mean temperature difference, contrast and homogeneity, among others. In 2018, Adam et al. [40] proposed a CAD system where the segmented images are submitted to discrete wavelet transform (DWT) and higher order spectra (HOS) techniques, and then several texture and entropy features are extracted from the coefficients. The extracted features are ranked using t-values and classified using a SVM classifier. In a subsequent work [41], they use a double density-dual tree-complex wavelet transform (DD-DT-CWT) to decompose the image. Several entropy and texture features are extracted from the decomposed images, namely, Hu’s invariant moment, gray level co-occurrence matrix, Shannon, Renyi, Kapur, Yager, Fuzzy, and Vadja. The features obtained were as follows: 4032 for the left foot, 4032 for the right foot, and 8064 for the bilateral foot. Saminathan et al. [42] segmented the plantar region by using region growing and extracted texture features to 11 regions within the foot. A symmetrical analysis was performed in such regions to classify them as either normal or ulcer. Maldonado et al. [43] performed a foot segmentation from the visible image by using DL, which was used afterwards to extract the plantar region in the thermogram. They classify the risk of ulceration or necrosis according to temperature differences.

### 2.2. Classification with Computational Intelligence Methods

The use of computational intelligence algorithms (CI) has been extended successfully in applications that involve pattern recognition, classification, automatic control and optimization, among others. Within the black-boxes methodologies, ANN is considered to be the most popular one, where the vast majority of the reported article reaches an accuracy of 80% in the prediction of DM. An alternative that has improved such level of accuracy is SVM, rising as the most successful algorithm in both biological and clinical datasets in DM [44]. The reason is that ANN uses derivative-based methods to update the weights, undergoing a slow convergence rate and often yielding suboptimal solutions [45]. Another outstanding methodology that has been gaining relevance is the use of massive neural structures to learn at several levels of abstraction; this is called Deep Learning. We will now mention some related works using CI methodologies in several tasks of DM’s foot classification. Kavakiotis et al. [44] present a systematic review of machine learning applications and data mining. The tasks include prediction, diagnosis, complications and health care, among others related to diabetes. After mentioning a series of different types of DM and examples of data, the paper compares algorithms, like Logistic regression (LR), ANN, Random Forest, and k-NN. We would like to emphasize that the accuracy of an algorithm depends on the type of data (dimensionality, origin, and kind); however, SVM is the most successful and widely used classifier. In [46], the work makes emphasis on efficient coding data by decreasing input data redundancy using independent component analysis algorithms (ICA). The results are obtained by testing the algorithm in the Pima Indians Diabetes database, where the SVM algorithm classifies the diabetics with a 98% accuracy rate. ICA improves the ability of the classifier by finding proper class boundaries; the reason is that ICA reduces the statistical dependence of the collected data. An exciting work mentioned in [47] goes beyond categorizing the types of DBs. The work tries to envisage the side-effects or other chronic diseases a patient should anticipate. For such purpose, the prediction/classification tasks consider more descriptive information composed of independent variables from former and current consultants of DM. Moreover, they add symptoms of the patients to the algorithms. Other works like [48] present an ensemble-type classifier, where algorithms like ANN, Naive Bayes, k-NN, and Random Forest, among others, are combined and perform better than all other individual counterparts.

All the works mentioned above and many others follow a crucial step for the automated analysis: the extraction of discriminant features from the images. This process is still done by human researchers. The real tendency is to let computers learn the features by themselves, trying to optimally represent the data for the problem at hand. This is the basic concept of many deep learning algorithms, where the massive structure of the ANN transforms the data while it learns increasingly higher level features. The tendency in the use of DL is to provide an increase in the accuracy level in classification processes; however, DL usually requires a large amount of data in the training process and the difficult task of labeling such training set. Some promising alternatives have emerged to deal with these drawbacks. There are two main ways to train DL architectures, either by training from scratch (or full training), which requires a large quantity of labeled training data, or by using extensive computational and memory resources, like Graphics Processing Units (GPUs), to obtain high velocities of processing. In the medical domain, this requirement may be difficult in the task of expert annotation and in the availability of patients to get a considerable number of images. A promising alternative is to fine-tune a pre-trained DL structure using a large labeled dataset from a different application. In the case of medical images, fine-tuning proved to either outperform or, in the worst case, perform as well as a Convolutional Neural Network (CNN) trained from scratch. The experiments considered four different medical imaging applications in three specialties: radiology, cardiology, and gastroenterology, involving classification and segmentation [49].

Litjens et al. present in [50] a work surveying the use of DL in medical image analysis. They study the implementation for image classification, object detection, segmentation, registration, and other tasks, mentioning its application in neural, retinal, pulmonary, digital pathology, breast, cardiac, abdominal, and musculoskeletal medical areas. The work presented in [51] proposes the use of traditional computer vision techniques in the detection of diabetic foot ulcers (DFU). They emphasize the importance of these methods, which represent a cost-effective, remote, and convenient healthcare solution over the traditional costly clinical approaches that rely on patient and clinical vigilance. Their proposal consists of improving the extraction of essential features for DFU classification based on a neural architecture, which is a combination of essential aspects of CNNs in their depth and parallel convolutional layers. The network is called diabetic foot ulcer network (DFUNet), and it reaches an accuracy of 94%, outperforming DL pre-trained structures like LeNet, AlexNet, and GoogleLeNet. The same author extends his research using DL in multi-class semantic segmentation for melanomas, benign lesions, and the pre-cancer stage called *seborrhoeic keratoses* [52].

Finally, we can conclude that, in the field of medical images, DL techniques are permeating and improving traditional processes, including the analysis, diagnosis, detection, classification, and segmentation. There are still challenges to be met, such as the automatic annotation to delineate and classify the images without the help of a specialist. Other issues include multi-class classification, as well as the improvement of the automatic classifiers in the detection, recognition, segmentation, and monitoring of the disease, among others.

## 3. Methodology

This section describes all the steps followed to obtain a final comparison of the machine learning-based classifiers mentioned above. For purposes of this work, 110 thermograms of DM subjects obtained from a public thermogram database were used [53].

In order to use the MLP and SVM algorithms, a process of selection of the region of interest (ROI) for segmentation is required in addition to the further extraction of relevant features. In this case, we use a histogram-based segmentation method represented by fuzzy sets and optimized with an evolutionary optimization technique. This process is mentioned briefly in this section, but it is expanded in the appendices at the end of the work. Finally, we briefly mention the description of the three machine learning-based classifiers and the new proposed DL structure.

### 3.1. Dataset and Data Augmentation

**Figure 1:**
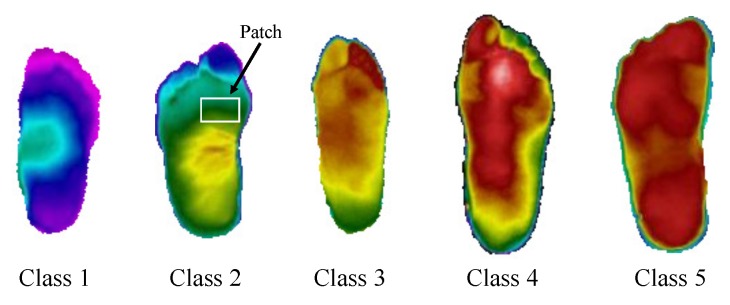
Images of the five level grades of the thermograms.

When DL structures are trained from scratch, they require a large number of images because of the enormous number of parameters trained in them. Data augmentation is an affordable technique to obtain such quantity of data when we do not dispose of it. Data augmentation consists of a combination of various processing techniques, like rotation, flipping, contrast enhancement, using different color, space, and random scaling. In this work, rotation is performed at angles of $90^{\circ },180^{\circ },$ and $270^{\circ }.$ We used three types of flipping (horizontal flip, vertical flip, and horizontal+vertical flip) performed on the original patches. We also obtained several patches of each image, allowing us to increase the data set tenfold. Figure 1 shows five classes of thermal change that can be found in the database and an example of the extracted patches.

### 3.2. Automatic Segmentation

**Figure 2:**
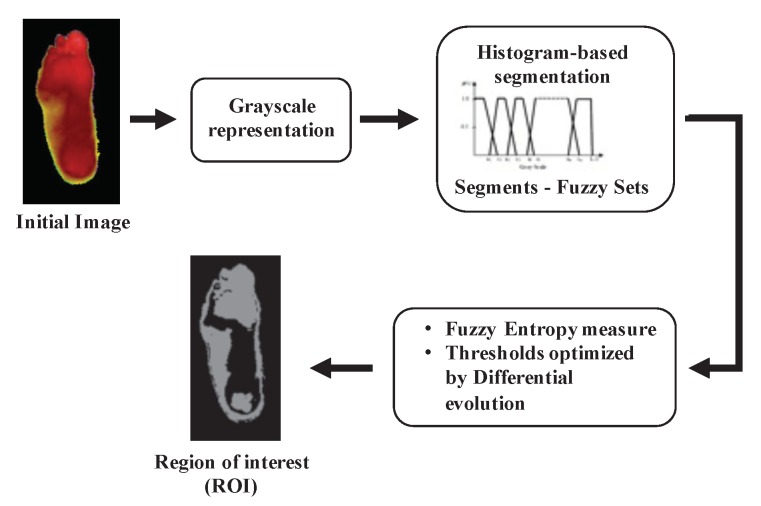
Automatic segmentation process [26].

The MLP and SVM require the extraction of relevant features to be introduced to the classifiers before training them. In this paper, the ROI of DM patients is segmented before the feature extraction using a histogram-based method. In the process of obtaining the partition of a digital image into multiple segments, a variety of image segmentation methods have been developed, such as thresholding, clustering-based methods, compression-based methods, histogram-based methods, and edge detection, among others. This work uses a histogram-based method, mainly using fuzzy logic, representing the segments of the image. The fuzzy logic approach for image processing allows us to use membership functions, defining the degree to which a pixel belongs to one segment or another. Furthermore, we obtain a better definition of segments by using fuzzy logic according to the measure of entropy. The optimized parameters are obtained by using a heuristic optimization technique based on Differential Evolution. The Appendices Appendix A and Appendix B contain an expanded explanation of this, and Figure 2 represents the basic steps of this process.

### 3.3. Machine Learning Classifiers

Computer aids have become an indispensable necessity in image-based medical decision-making processes, such as in the detection and diagnosis, automated segmentation, automated image annotation, and image retrieval [54]. The use of computational intelligence algorithms tries to overcome some drawbacks, including the exhaustive task of interpreting a large number of images. Other algorithms involve achieving more accurate diagnosis systems, thereby providing a higher level of reliability, which the patient needs. The advance of medical imaging technologies, which provide new imaging modalities and methodologies, require new computational algorithms according to the characteristics of the images and the high quantity of handled data. Some of the most widely used algorithms are ANN and SVM. However, DL has been used in the classification of medical images as well as in other fields, as a result of the high accuracy rate obtained from its complex learning structure, and, in many cases, the vast quantity of data processed, which allows these structure to obtain multiple levels of feature abstractions from the data. In this work, the former two algorithms are compared with DL, presenting the advantages and disadvantages of their use. For a better explanation of these algorithms, please refer to the sections of the appendix.

### 3.4. Multiple Classes

**Figure 3:**
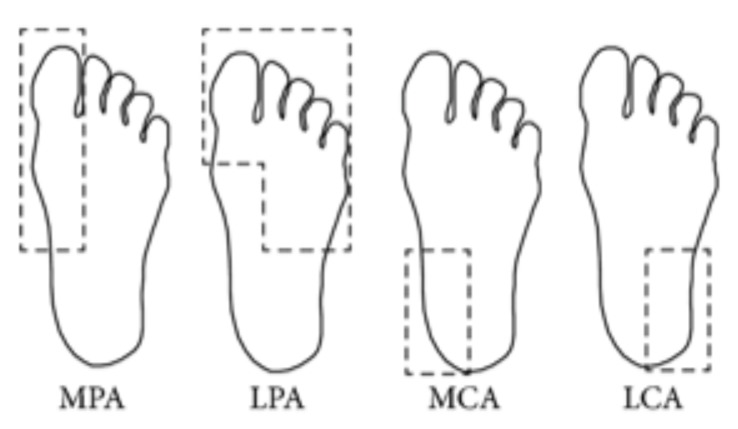
Angiosomes suggested by Taylor and Palmer [55] for temperature analysis.

Previous research of some authors in this work showed that it is possible to estimate local plantar temperatures based on the angiosome concept [19]. An angiosome is a composite unit of tissues supplied by an artery, providing valuable information relating temperature data to artery damage. For this purpose, the foot is divided into four angiosomes: medial plantar artery (MPA), lateral plantar artery (LPA), medial calcaneal artery (MCA), and lateral calcaneal artery (LCA). The obtained information using the angiosomes is related not only to the damage generated by DM in arteries but also to the associated ulceration risk since it is used to compute local temperatures. See Figure 3 for the illustration of the angiosomes.

Additionally, they proposed a new thermal change index (TCI) as an attempt to overcome some drawbacks in the measurement of thermal measure-based algorithms in the diabetic foot. These include differences in thermal pattern assumptions when both feet are compared, which occur, for instance, when the patient presents a partial or total amputation or ulceration. TCI takes advantage of the Control Group (CG) well-established butterfly pattern that is used individually to compare each foot affected by the disease. The TCI value is the mean difference between corresponding angiosomes from a DM subject and the reference values obtained from angiosomes of the CG:

(1) $$TCI=\frac{\sum \left|C_{ang}-DM_{ang}\right|}{4}$$

where $C_{ang}$ and $DM_{ang}$ are the temperature values of the angiosome for the control group and for a DM subject, respectively.

Five categories of the change degree of the plantar regions were obtained based on the results in [19]. These are used in this work to test the classification algorithms with multiple-classes of the DM-thermal images.

### 3.5. Performance Evaluation and Classification Scheme

In all our experiments, we report Sensitivity, Specificity, Precision, Accuracy, F-measure, and Area under the curve (AUC) as our evaluation metrics. It is well known that, in medical imaging, Sensitivity and Specificity are relevant metrics to evaluate classifier completeness [56]:

(2) $$Sensitivity=\frac{TP}{TP+FN}$$

(3) $$Specificity=\frac{TN}{FP+TN}$$

(4) $$Precision=\frac{TP}{TP+FP}$$

(5) $$Accuracy=\frac{TP+FN}{TP+TN++FP+FN}$$

(6) $$F_{measure}=\frac{2*TP}{2*TP+FP+FN}$$

### 3.6. Proposed Deep Learning Network

After not being able to obtain satisfactory results with SVM, MLP, AlexNet, and GoogLeNet, especially with the five levels of classification, we propose a new type of DL structure. The name of this network is Diabetic Foot Thermograms Network (DFTNet). With this proposal, we considerably reduce the number of layers, compared with the 22 layers of GoogLeNet, which also result in a decreasing training time.

The parameters used from training the DFTNet are a maximum of 100 epochs, a minibatch size of 64, and the Adam solver with a learning rate of 0.001. The configuration of the computer is: CPU Intel i7–7700 HQ @2.8 GHz, GPU NVIDIA GeForce GTX 1060, RAM 16 GB, Software Matlab. The structure of DFTNet is shown in Table 1.

## 4. Experimental Results

We divide the experimental results into two parts. The first part presents the results of the process of automatic segmentation, using the measure of fuzzy entropy and optimizing with meta-heuristics like DE. We show the relevant results from this procedure that let us obtain the best region of interest (ROI). The second part presents a comparison of the most pertinent classifiers in the area of computational intelligence; these are ANN, SVM, and DL. The first two require the additional stage, which involves segmenting the ROI and extracting relevant features, whereas the last one “learns” directly from the dataset.

### 4.1. Automatic Segmentation with DE

As mentioned earlier, diabetic foot classification follows an automatic segmentation to obtain the ROI. In this case, DE is used as an optimization procedure of the fuzzy entropy measure. Once a normalized histogram represents the image with L=255 gray levels, the segmentation levels determining the thresholds are optimized with DE. The objective is to obtain the maximum value of the total fuzzy entropy function (See Appendix), where each *n*-level segment is represented by fuzzy sets and its corresponding membership function values. After the optimal value of entropy is reached, the n−1 number of thresholds is extracted from the fuzzy parameters.

In our case, the ROI for a healthy subject as part of the control group (CG) is given in the Butterfly Pattern distribution, which presents the highest temperature in the arch, and decreases as it moves away from this area. In the case of the DM group, this distribution of highest temperatures defining the ROI is shown in different regions of the foot [18]. In the histogram of the thermogram with 32 gray levels, the global maximum represents the image background, while the other represents the plantar region (PR). Thus, by using thresholding, which is one of the most common pixel-based methods to segment an image [57], the ROI is obtained for posterior classification with three of the most common and relevant classifiers.

Following the procedure of automatic segmentation, several thresholds are considered to maximize the proposed fuzzy entropy measure, and, at the same time, obtain the desired pattern, distinguishing the CG from the DB group. We can consider that, for each proposed number of thresholds (n−1), we will have *n*-level segments or classes.

**Figure 4:**
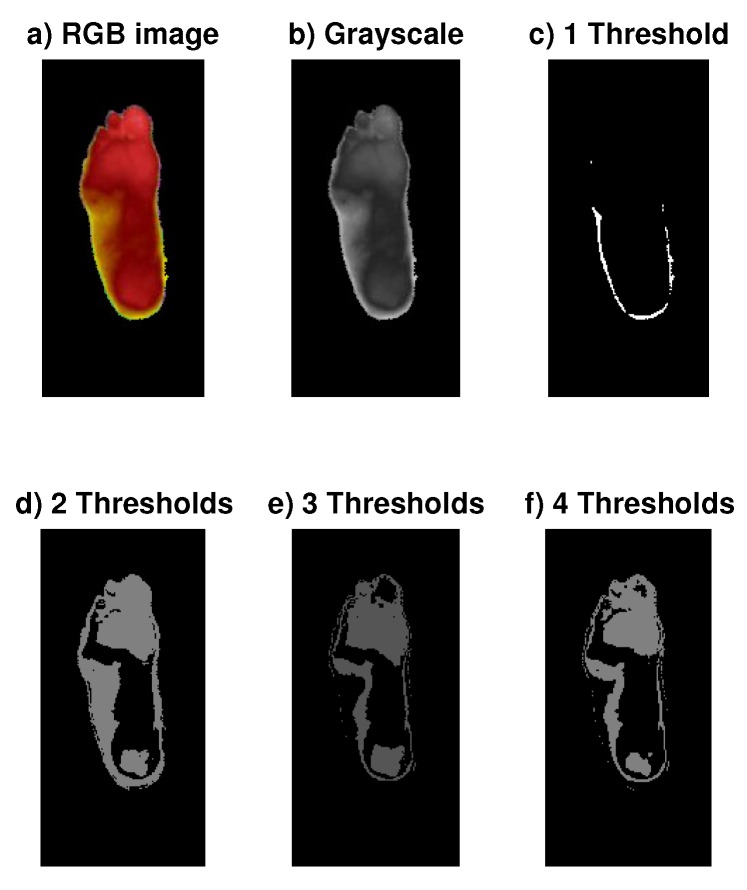
Images of the automatic segmentation process of one case of the DM group, including the RGB original image, the grayscale representation, and the obtained images from one to four thresholds [26].

For instance, Figure 4 shows the results of one segmented image of the DB group for two, three, and four thresholds. The entropy values obtained were 0.78106, 0.52698, and 0.45723, respectively. We also tried the one-threshold case, defining two segments or classes, obtaining the highest level of entropy: 0.96938. However, the segmented pattern does not satisfy a defined pattern for the ROI. Thus, our criteria lie between having a high value of entropy and obtaining a defined ROI pattern at the same time.

**Figure 5:**
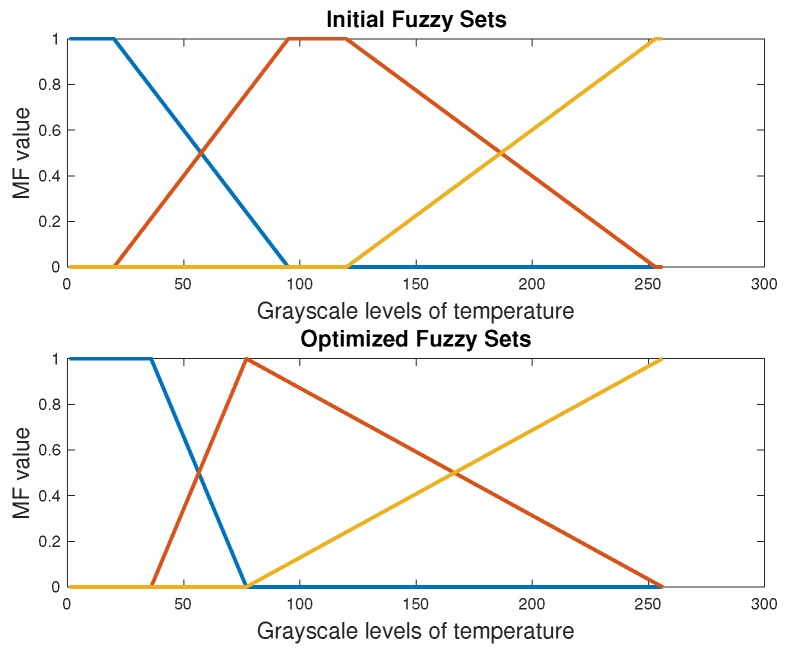
Fuzzy sets of the segmentation process in a DM foot with the optimal value of two thresholds [26].

**Figure 6:**
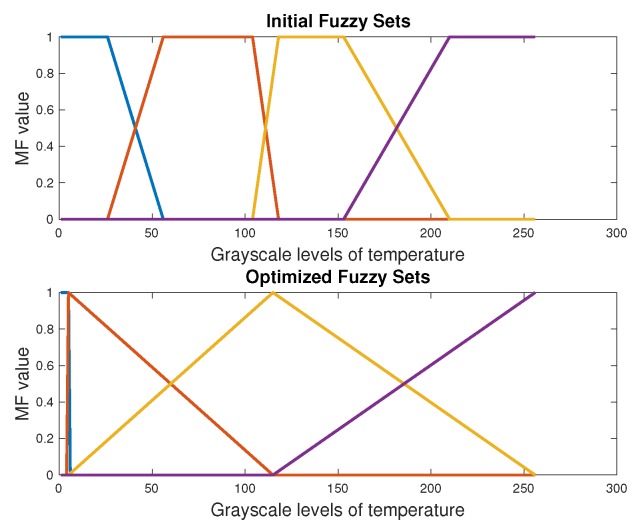
Fuzzy sets of the segmentation process in a CG foot with the optimal value of three thresholds [26].

This automatic segmentation based on DE defines the thresholds of fuzzy sets. Figure 5 and Figure 6 show the initial and final fuzzy sets after the DE-optimization process for one case of the DM and CG, respectively. The location of the selected thresholds is in the middle part of the overlap of fuzzy sets. We can observe the modification not only in the parameters and form of the fuzzy sets by the DE, but also in the location of the thresholds. The initial thresholds are located in $T_{1}=64$ and $T_{2}=243$ within the 255 values of the histogram. After the evolutive optimization, these values are: $T_{1}=56$ and $T_{2}=167$. The next section presents the results of classification with SVM and ANN. The extracted features for these classifiers were obtained with this automatic segmentation process.

### 4.2. Classification of Multiple Classes

For multi-class classification (see Section 3.4), three different machine learning algorithms were used: MLP, SVM, and DL. The first two machine learning classifiers require an extracted vector of features from the segmented images by the DE histogram-based algorithm, whereas DL does not need this step due to its massive structure. The extracted feature’s vector for the first two algorithms contains five elements per image, and these are related to the segmented area: number of pixels, mean value, variance, maximum entropy value, and the index value.

We begin by presenting the results of ANN and SVM, which share a similar classification procedure in the sense that they use extracted features from the ROI. The training process of both techniques requires a split of the training set into 85% for training, 5% for validation, and 10% for testing. We adopted the 10-fold cross-validation technique. Both algorithms run on Matlab. The results of the MLP and SVM with the required measures are shown in Table 2. The best results were obtained with the 3rd segmentation level. This is why we only present these results. In addition, the best results were achieved using a 3-layer ANN structure and a linear kernel for the SVM classifier.

In these cases, the multiple classes are obtained from the TCI-values. The classification is a bit complicated because of the similarities in thermograms among DM-patients. Regarding ANN and SVM, SVM has some advantages in improving some performance measures due to the high-dimensional mapping and the use of nonlinear kernels, enhancing the classification accuracy.

In addition, we use two pre-trained DL models (AlexNet and GoogLeNet), and our proposed DL-structure, the DFTNet. AlexNet is a convolutional neural network that has been trained on over a million images and can classify images into 1000 object categories (such as a keyboard, coffee mug, pencil, and many animals) [58]. The training set is divided into 70% for training and 30% for validation. AlexNet has five convolutional layers and three fully connected layers. The fine-tuning process in our case consists of extracting the last three layers that were previously configured to classify 1000 images and to be re-trained with our data set of the thermal images of diabetic and control groups. We replace these three layers by a fully connected layer, a soft-max layer, and a classification output layer. The first layer requires input images of 227-by-227-by-3, where 3 is the number of color channels. In addition, the process requires additional augmentation on the images: performing operations like randomly flipping along the vertical axis and randomly translating them up to 30 pixels, horizontally and vertically. This step of data augmentation prevents the network from overfitting and memorizing the exact details of the training images.

GoogLeNet is another state-of-the-art CNN architecture with 22 deep network layers. GoogLeNet proposes a new module called inception, which acts as a multiple convolution filter input, and it is processed on the same input while doing pooling at the same time. All the outcomes are merged into a single feature layer, allowing the model to take advantage of multiple level feature extraction from each input [59].

The performance measures of the pre-trained AlexNet and GoogLeNet models and those obtained by our proposed DFTNet model, which was trained from scratch, are shown in Table 3 and Table 4, respectively. A 10-fold cross-validation technique was used for each case.

We achieved some good results in DL-structures with well-separated classes like 1-5, 2-5, 4-1, and so on. However, neighboring classes, especially 3-4, present the lowest values in precision, accuracy, and other measures. The problem is that, in neighboring classes, and especially in classes like 3-4 and 4-5, the patterns are so similar and the training images do not provide sufficient information to learn in the DL-structures.

## 5. Discussion

Two traditional machine learning classifiers and three models based on CNNs were used for classification of the thermograms. We present a five-level classification procedure, where the thermograms are divided into five classes according to the TCI mentioned in Section 3.4.

**Figure 7:**
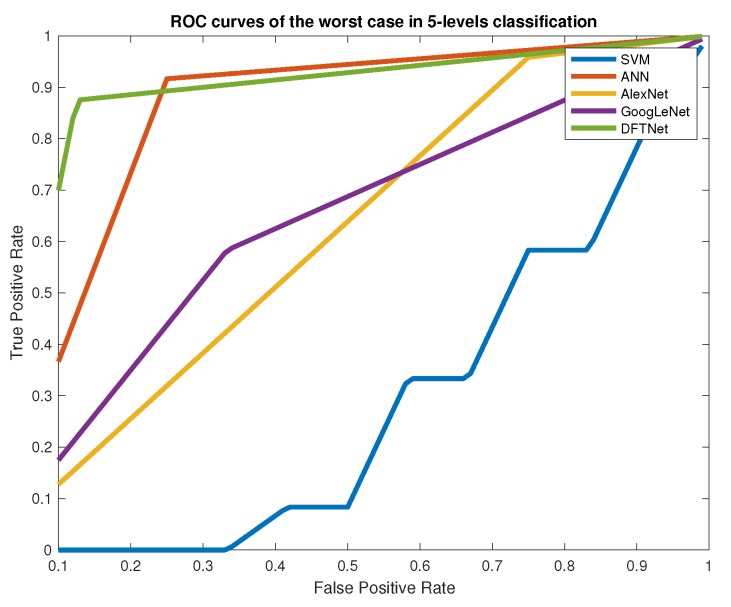
ROC curves of the worst 5-level classification case (Classes 3 and 4).

The first two classifiers required an additional step of feature extraction, obtaining the ROI with the histogram-based segmentation procedure, which uses a fuzzy entropy measure and optimization of the segments with DE. The extracted feature vector contains relevant information from the thermogram’s ROIs, like the number of pixels, mean value, variance, maximum entropy value, and the index value of the grayscale (see Figure 4). This step is a little bit cumbersome due to the optimization procedure and the attempt with different thresholds in the segments to obtain the best ROIs. It is a method that involves more the participation of the expert. In regard to the dataset comments, the number of samples is not large, and we did not even increase the number of samples by dividing each image into patches. The CNN classifiers require the additional step of data augmentation for the three structures (GoogLeNet, AlexNet, and DFTNet). This is why we chose to divide each image into patches, like the one shown in Figure 1, increasing our data set about ten times in addition to the data augmentation process of the CNN structures. The classification is carried out comparing one-vs.-one classes (OVO) to use regular performance measures. We obtained the highest values in sensitivity, specificity, AUC, and Accuracy with classifiers like SVM, and, in the CNN structures, in almost all the pairs of compared classes, especially in well-separated classes like 1-3, 1-4, 1-5, 2-4, 2-5, 3-5, etc. In consecutive classes, the best results were obtained in cases 1-2 and 4-5. Because distributions without thermal change are found in class 1, the transition to class 2 is important as this may indicate the initial step in a thermal change. This transition is remarked by the loss of the butterfly pattern with slightly higher temperatures [19]. However, classes 3 and 4 present the lowest values of performance measures due to the similarities between them. In these two classes, we can find larger hot regions, but the difference is not as obvious as in the transition to class 5, where the hot regions already cover almost the entire plantar region. Figure 7 shows the ROC curves for this worst case of comparison between classes 3 and 4. In this case, SVM achieved the lowest values of AUC and other measures, like sensitivity and accuracy. The two highest results are of our DFTNet and the common ANN, with AUC-values of 0.8533 and 0.8333, respectively. ANN has 0.66 in sensitivity and 0.833 in accuracy, while DFNet has 0.9167 in sensitivity and 0.853 in accuracy. We can attribute the highest results of our DFNet to the specific design of the network to this type of images. Although the structures of GoogLeNet and AlexNet are more complex and are supposedly better classifiers, they were trained with a different type of images.

In general, we can comment that CNNs have advantages when they are trained with a sufficient number of images if they are trained from scratch. The other option is using pre-trained networks, but it works well if datasets are not too difficult to classify. In this research, the similarities between classes when we classified the five levels made it difficult for the pre-trained CNNs and SVM. In addition, dividing the images into five levels using the TCI values decreases the number of samples for each class, which is not optimal for the classifiers, especially for the CNNs. The tactics of dividing each image into patches selected by the expert, along with the operations of data augmentation for CNNs and the design of our own CNN structure, provided the best results for the easiest and worst cases of classification.

## 6. Conclusions

This work presents a comparison of conventional classifiers like ANN, SVM, and those of current importance like CNNs. The aim is to classify patterns in thermal images of patients with DM. The work includes the classification of five levels within DM-patients. The results of the first simulations using traditional ANN and SVM classifiers obtained satisfactory results after a feature extraction process. However, the tendency of using DL structures is not only to gain an increase in the accuracy of the classification, but also to avoid, in some cases, the exhaustive task of feature extraction and segmentation of the desired patterns. An advantage of supporting our work in the use of such DL structures is the use of pre-trained networks. The results obtained with DL are better and have low training time.

We can observe in the results that consecutive classes were affected by similarities in temperature distributions of the thermograms. In this case, CNNs like GoogLeNet and AlexNet obtained the same accuracy results, which were not satisfactory. We proposed a new design of CNN with a simple structure but a better design. The proposed DFTNet lets us obtain satisfactory results using measures of sensitivity, specificity, accuracy, and AUC-values, among others, even in the worst cases of classification.

As future work, we are working on obtaining more images of thermograms, and improving the structure of our CNN, perhaps with parallel convolution operations, like the GoogLeNet. We aim to get better results of classification with less participation from the expert in the selection of patches and ROIs.

This research is aimed to classify thermograms and predict the presence of ulceration in feet. Another extension of this work could be to classify feet with ulcerations, which contain quite distinctive texture and color features when compared with normal healthy skin.

## Figures and Tables

**Table 1:** Description of the DFTNet Architecture.

Layer No.	Layer Type	Filter Size	Stride	No. of Filters	FC Units
Layer 1	Conv.	7 × 7	1 ×1	32	-
Layer 2	Max-Pool	3×3	2×2	-	-
Layer 3	Conv.	1×1	1×1	64	-
Layer 4	Conv.	3×3	1×1	64	
Layer 5	Max-Pool	3×3	2×2	-	-
Layer 6	Conv.	3×3	1×1	32	
Layer 7	Max-Pool	2×2	2×2	-	-
Layer 8	Conv.	3×3	1×1	32	-
Layer 9	Full Conn.	-	-	-	No. Classes

**Table 2:** The performance measures of SVM (left) and ANN (right) in classification of the five classes.

Case	1st Class	2nd Class	Sensitivity	Specificity	Precision	Accuracy	F-measure	AUC
1	1	5	1.0000–0.9167	1.0000–0.9167	1.0000–0.9167	1.0000–0.9167	1.0000–0.9167	1.0000–0.9167
2	2	5	1.0000–0.9167	1.0000–0.8333	1.0000–0.8462	1.0000–0.8750	1.0000–0.8800	1.0000–0.8750
3	1	4	0.9167–0.6667	1.0000–1.0000	1.0000–1.0000	0.9583–0.8333	0.9565–0.8000	0.9167–0.8333
4	3	5	1.0000–1.0000	0.9167–0.5833	0.9231–0.7059	0.9583–0.7917	0.9600–0.8276	0.9826–0.7917
5	2	4	0.9167–0.6667	0.5000–0.9167	0.6471–0.8889	0.7083–0.7917	0.7586–0.7619	0.9167–0.7917
6	1	3	1.0000–0.5000	1.0000–1.0000	1.0000–1.0000	1.0000–0.7500	1.0000–0.6667	1.0000–0.7500
7	4	5	1.0000–1.0000	0.9167–0.4167	0.9231–0.6316	0.9583–0.7083	0.9600–0.7742	1.0000–0.7083
8	3	4	0.2500–0.6667	0.5833–1.0000	0.3750–1.0000	0.4167–0.8333	0.3000–0.8000	0.3194–0.8333
9	2	3	1.0000–0.8333	1.0000–1.0000	1.0000–1.0000	1.0000–0.9167	1.0000–0.9091	1.0000–0.9167
10	1	2	0.8333–0.5000	0.5833–1.0000	0.6667–1.0000	0.7083–0.7500	0.7407–0.6667	0.7639–0.7500
**Average**			0.8917–0.7667	0.8500–0.8667	0.8535–0.8989	0.8708–0.8167	0.8676–0.8003	0.8899–0.8167

**Table 3:** The performance measures of AlexNet (left) and GoogleNet (right) in classification of the five classes.

Case	1st Class	2nd Class	Sensitivity	Specificity	Precision	Accuracy	F-measure	AUC
1	1	5	0.9545–0.9091	1.0000–1.0000	1.0000–1.0000	0.9783–0.9565	0.9767–0.9524	0.9773–0.9545
2	2	5	1.0000–0.9583	0.9583–0.9583	0.9600–0.9583	0.9792–0.9583	0.9796–0.9583	0.9792–0.9583
3	1	4	0.7727–0.7727	1.0000–0.9583	1.0000–0.9444	0.8913–0.8696	0.8718–0.8500	0.8864–0.8655
4	3	5	0.9583–0.9167	0.7917–0.7917	0.8214–0.8148	0.8750–0.8542	0.8846–0.8627	0.8750–0.8542
5	2	4	0.9167–0.7917	0.8333–0.9167	0.8462–0.9048	0.8750–0.8542	0.8800–0.8444	0.8750–0.8542
6	1	3	0.9545–0.9091	0.5417–1.0000	0.6563–1.0000	0.7391–0.9565	0.7778–0.9524	0.7481–0.9545
7	4	5	0.7500–0.8333	0.8750–0.7500	0.8571–0.7692	0.8125–0.7917	0.8000–0.8000	0.8125–0.7917
8	3	4	0.5000–0.5833	0.5417–0.6250	0.5217–0.6087	0.5208–0.6042	0.5106–0.5957	0.5208–0.6042
9	2	3	0.8333–0.4583	0.6250–0.9583	0.6897–0.9167	0.7292–0.7083	0.7547–0.6111	0.7292–0.7083
10	1	2	0.8182–0.3182	0.6250–0.9167	0.6667–0.7778	0.7174–0.6304	0.7347–0.4516	0.7216–0.6174
**Average**			0.8458–0.7451	0.7792–0.8875	0.8019–0.8695	0.8118–0.8184	0.8171–0.7879	0.8125–0.8163

**Table 4:** The performance measures of our proposed DFTNet structure classifying the five classes.

Case	Class 1	Class 2	Sensitivity	Specificity	Precision	Accuracy	F-measure	AUC
1	1	5	1	1	1	1	1	1
2	2	5	1	1	1	1	1	1
3	1	4	1	0.9583	0.9565	0.9783	0.9778	0.9792
4	3	5	0.8333	0.9583	0.9524	0.8958	0.8889	0.8958
5	2	4	1	1	1	1	1	1
6	1	3	0.9545	0.9583	0.9545	0.9565	0.9545	0.9564
7	4	5	0.9583	0.9583	0.9583	0.9583	0.9583	0.9583
8	3	4	0.9167	0.75	0.7857	0.8333	0.8462	0.8533
9	2	3	0.9167	0.875	0.88	0.8958	0.898	0.8958
10	1	2	0.9545	0.9167	0.913	0.9348	0.9333	0.9356
**Average**			0.9534	0.9375	0.9401	0.9453	0.9457	0.9455

**Figure A1:**
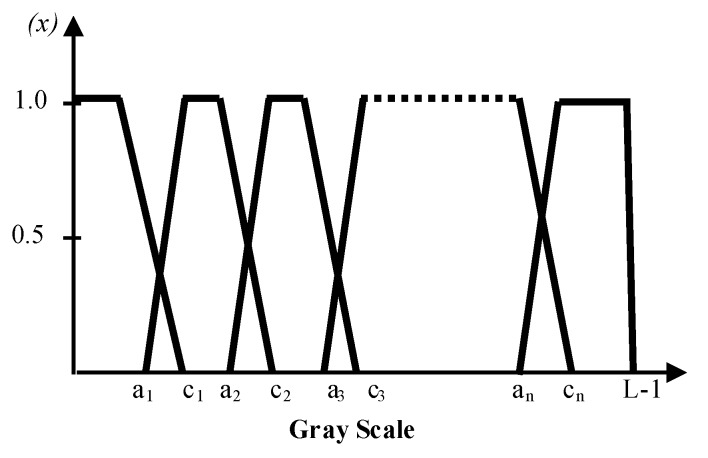
Fuzzy membership function for *n*-level thresholding [26].

## Supplementary Materials

The proposed DFTNet is available online at https://www.mdpi.com/1424-8220/20/6/1762/s1.

## Appendix A. Segmentation: Multilevel Shannon Entropy and Fuzzy Entropy

Regarding Information Theory, entropy is the measure of the amount of information that is missing before the reception of a message (information) [60]. Information entropy measures the average amount of information in a message in terms of discrete probabilities $p_{i}$.

In terms of image segmentation, the concept of Shannon entropy is based on the maximum amount of information concerning the distribution of the target and background of the image. By measuring the entropy of the gray histogram of the image, the best threshold is found [61]. Thus, the entropy of the total image is defined as follows:

$$H\left(I\right)=-\sum_{i=1}^{n}p_{i}\log_{2}p_{i}$$

where $p_{i}$ is the probability of occurrence of the i-th gray level, and *I* denotes an 8-bit gray level image of dimension M×N. *P* is the normalized histogram for the image with L=255 gray levels. In this case, we obtain the n−1 thresholds (*t*) partitioning the normalized histogram into *n* classes, that is, by maximizing the total entropy. This measure constitutes individual measures of entropy for each one of the proposed classes in the image [62].

However, a more precise measure of entropy that deals with imprecise, vague, or noisy data relies on Fuzzy Logic, having fuzzy sets as essential elements, and the corresponding membership value within such sets.

Fuzzy sets are a generalization of classical sets, that is, instead of using a sharp definition of belongingness of an element to a certain set, such element can partially belong to this set and another with certain degrees. A fuzzy set is defined as $A=\left\{\left(x,\mu_{A}\left(x\right))|x\in X\right)\right\}$, where $0<\mu_{A}\left(x\right)<1$ is the membership function measuring the closeness (or belongingness) of *x* to *A*.

In this case, fuzzy sets represent the segmented regions defining the image classes. For instance, using trapezoidal membership functions, the estimation of the membership parameters of the *n* segmented regions, $\mu_{1},\mu_{2},\ldots ,\mu_{n}$ for the *n*-level thresholding, namely $a_{1},c_{1},\ldots ,a_{n-1},c_{n-1}$, where $0\leq a_{1}\leq c_{1}\leq ,\ldots ,\leq a_{n-1}\leq c_{n-1}\leq L-1$. See Figure A1.

According to [63], the membership function for the *n*-level thresholding is:

$$\mu_{1}\left(k\right)=\left\{\begin{array}{cc} 1 & k\leq a_{1}\\ \frac{k-c_{1}}{a_{1}-c_{1}} & a_{1}\leq k\leq c_{1}\\ 0 & k>c_{1}\\ \; & \vdots \end{array}\right.$$

$$\mu_{n-1}\left(k\right)=\left\{\begin{array}{cc} 0 & k\leq a_{n-2}\\ \frac{k-a_{n-2}}{c_{n-2}-a_{n-2}} & a_{n-2}<k\leq c_{n-2}\\ 1 & c_{n-2}<k\leq a_{n-2}\\ \frac{k-c_{n-1}}{a_{n-1}-c_{n-1}} & a_{n-1}<k\leq c_{n-1}\\ 0 & k>c_{n-1} \end{array}\right.$$

$$\mu_{n}\left(k\right)=\left\{\begin{array}{cc} 1 & k\leq a_{n-1}\\ \frac{k-a_{n}}{c_{n}-a_{n}} & a_{n-1}\leq k\leq c_{n-1}\\ 1 & k>c_{n-1} \end{array}\right.$$

The maximum fuzzy entropy for each of the *n*-level segments is:

$$\begin{array}{c} H_{1}\left(t\right)=-\sum_{i=0}^{L-1}\frac{p_{i}*\mu_{1}\left(i\right)}{P_{1}}\ln\frac{p_{i}*\mu_{1}\left(i\right)}{P_{1}}\\ H_{2}\left(t\right)=-\sum_{i=0}^{L-1}\frac{p_{i}*\mu_{2}\left(i\right)}{P_{2}}\ln\frac{p_{i}*\mu_{2}\left(i\right)}{P_{2}}\\ H_{n}\left(t\right)=-\sum_{i=0}^{L-1}\frac{p_{i}*\mu_{n}\left(i\right)}{P_{n}}\ln\frac{p_{i}*\mu_{n}\left(i\right)}{P_{n}} \end{array}$$

where

$$\begin{array}{c} P_{1}\left(t\right)=\sum_{i=0}^{L-1}p_{i}*\mu_{1}\left(i\right);P_{2}\left(t\right)=\sum_{i=0}^{L-1}p_{i}*\mu_{2}\left(i\right);\cdots ;\\ P_{n}\left(t\right)=\sum_{i=0}^{L-1}p_{i}*\mu_{n}\left(i\right) \end{array}$$

Maximizing the total entropy allows us to obtain the optimal value of parameters:

$$\begin{array}{c} \phi (a_{1},c_{1},\ldots ,a_{n-1},c_{n-1})=\\ Argmax(\left[H_{1}\left(t\right)+H_{2}\left(t\right)+\ldots +H_{n}\left(t\right)\right]) \end{array}$$

Then, extracting the n−1 number of thresholds from the fuzzy parameters as follows:

$$t_{1}=\frac{a_{1}+c_{1}}{2};t_{2}=\frac{a_{2}+c_{2}}{2};\cdots t_{n-1}=\frac{a_{n-1}+c_{n-1}}{2}.$$

The purpose of this work is to modify these parameters, trying to obtain an optimal segmentation of the image. In this work, we use heuristic optimization based on differential evolution.

## Appendix B. Differential Evolution

Evolutionary computing (EC) draws inspiration from the process of natural evolution. A common task in the use of EC algorithms is the optimization of specific parameters of the proposed model, delivering the correct output for each known input [64]. Some of the existing techniques in EC include genetic algorithms (GA) or particle swarm optimization (PSO). We use differential evolution (DE), which has been shown to outperform GA and PSO for multilevel thresholding based image segmentation problems. Sarkar et al. [62,65] presented a comparison between these algorithms in terms of computational time, mean objective value, and standard deviation. The results favor DE in both visual and statistical comparisons of the segmented images.

The main idea with DE is that, given a population of candidate solution vectors in $R_{n}$, the *i*-th individual (parameter vector) of the population at generation (time) *t* is a *D*-dimensional vector containing a set of *D* optimization parameters:

$${\bar{X}}_{i}\left(t\right)=\left[x_{1},x_{2},\ldots x_{D}\right]$$

A change in the members of the population is performed by the creation of a donor vector $\bar{P}$. In this work, the donor vector $\bar{P}$ for each *i*-th member is created using the DE/rand/1 variant [66], that is, it is created by randomly choosing three other parameter vectors ($\left(r_{1},r_{2},r_{3}\right)\in \left[1,NP\right]$), and $r_{1}\neq r_{2}\neq r_{3}$. NP is the number of population members. Then, $\bar{P}$ is obtained by multiplying a scalar number *F* with the difference of any two of the three $r_{i}$ as follows:

$${\bar{P}}_{i,j}=X_{r_{1},j}+F\cdot \left(X_{r_{2},j}-X_{r_{3},j}\right)$$

where *j*-th is the component of the *i*-th vector [66]. Henceforth, for simplicity of the equations, we have omitted the use of *t*. A binomial crossover operation is used to increase the potential diversity of the population. The binomial crossover is performed for each of the *D* variables, subject to the crossover parameter $C_{r}\in \left[0,1\right]$. In addition, the number of parameters inherited from the mutant has a (nearly) binomial distribution. Thus, for each target vector ${\bar{X}}_{i}$, a trial vector ${\bar{R}}_{i}$ is created as follows:

$${\bar{R}}_{i,j}=\left\{\begin{array}{cc} P_{i,j} & \mathrm{if}\;rand_{i}\left(0,1\right)\leq C_{r}\\ X_{i,j} & otherwise \end{array}\right.The$$

where rand is a number in a uniform random distribution. Finally, the selection is performed to determine which one, whether the target vector or the trial vector, will survive in the next generation. If the trial vector yields a better value of the fitness function, it replaces the target vector in the next generation; otherwise, the parent remains without change:

$${\bar{X}}_{i,j}=\left\{\begin{array}{cc} {\bar{R}}_{i,j} & \mathrm{if}\;f\left({\bar{R}}_{i,j}\right)>f\left({\bar{X}}_{i,j}\right)\\ {\bar{X}}_{i,j} & otherwise \end{array}\right.$$

where f(·) is the fitness function.

### Appendix B.1. Artificial Neural Networks

The ability to learn from input data, with or without a teacher, has prioritized ANN as one of the primary tools in classification, segmentation, regression, and extraction of outstanding characteristics. A common ANN type for pattern recognition is the Multilayer Perceptron (MLP) feed-forward neural network. The success in the learning process of an ANN is related mainly to two aspects: the ANN architecture and the learning algorithm. For this case, the MLP is trained using the error-back-propagation algorithm. This is based on a generalized delta learning rule as an iterative gradient-based algorithm that minimizes the root-mean-squared error between the actual output of the ANN and the training set or desired output. The learning process consists of two steps. The first step is the feed-forward step, without modifications to the network’s parameters, that is: (1) Given a vector of characteristics $\vec{X}=\left[x_{1},x_{2},\ldots ,x_{N},\right]$ defining the ANN-input, it is pondered by a vector $\vec{W}$ of weights between neurons; (2) The induced local field of the ANN is produced by summing the products of the previous step, that is: $v=\sum \vec{W}\vec{X}$; (3) Finally, the ANN-output is computed as y=ϕ(v), where ϕ is the activation function of the neuron.

The second step, which is the back-forward step, modifies $\vec{W}$, in such a way that the error function $\epsilon =\frac{1}{N}\sum_{k=1}^{n}e{\left(k\right)}^{2}$ is minimized, e(k)=d(k)−y(k), and *N* is the number of samples. In general, the weights’ update follows a gradient descend learning rule, which is also called delta-rule:

$$w\left(k+1\right)=w\left(k\right)-\eta \frac{\partial E}{\partial W}$$

where η is the learning rate. Some other algorithms that improve the convergence in the learning process have been proposed. One of these is the called Levenberg–Marquardt.

### Appendix B.2. Support Vector Machines

Support Vector Machines (SVMs), like MLP and radial-basis-function networks, are another category of universal feed-forward networks. They are usually used for pattern classification and nonlinear regression [67]. SVMs like the MLP provide a hyperplane between two classes, functioning as a decision surface, but with the difference that SVMs maximize the margin of separation between positive and negative examples of such classes. Considering the training example ${\left(x_{i},y_{i}\right)}_{i=1}^{N}$, where $x_{i}$ is the input pattern, and $y_{i}=\pm 1$ the target output, the equation of the decision surface in the form of a hyperplane is:

$$f\left(x\right)=w^{T}x+b=0$$

where w is a weight vector and *b* is a bias. This best separating hyperplane is the result of finding w and *b*, minimizing $∥w∥$, such that, for all points ${\left(x_{i},y_{i}\right)}_{i=1}^{N}$,

$$y_{i}f\left(x_{i}\right)\geq 1$$

The support vectors are the $x_{i}$ on the boundary which $y_{i}f\left(x_{i}\right)=1$. The problem of minimizing $∥w∥$ is treated as a quadratic programming problem, obtaining the optimal solution by means of a Lagrange formulation [68].

In cases of nonlinearly separable data, SVMs allow the construction of hyperplanes in high dimensional feature spaces, retaining all the simplicity of an SVM-separating hyperplane. This is possible by using kernels and the mapping to these high dimensional spaces with inner-products (dot products). Depending on the kernel of the inner-product, different learning machines like polynomials, Gaussians and sigmoids functions, among others, characterize different nonlinear decision surfaces. The improvement in the performance of an SVM is by selecting the features that match a particular kernel, choosing an appropriate kernel that fits the features, or linearizing the features to a higher dimensional space and matching them with linear kernels [69].

### Appendix B.3. Deep Learning

Deep learning (DL) are computational models composed of multiple processing layers. These models are characterized by learning data representations with various levels of abstraction and discovering complex structures in large datasets. DL methods have dramatically improved the state-of-the-art in speech recognition, visual object recognition, object detection, and genomics, among others [70]. In this work, we use a Convolutional Neural Network (CNN) that is a subtype of deep discriminative architecture with satisfactory performance in processing two-dimensional data with grid-like topologies, such as images and videos [71].

In CNNs, the convolutional layers carry out the detection of certain local features in all the locations of their input images. Each node connects to only a small subset of spatially connected neurons in the input image channels. The sets of connection weights shared between the nodes, namely the kernels, enable the search for the same local features in the input channels. A convolutional layer has *n* kernels that learn to detect *n* local features, whose strength across the input images is visible in the *n* resulting feature maps. The pooling layer follows the convolutional layer and is used in order to reduce the computational complexity to achieve a hierarchical set of image features. CNNs contain several pairs of convolutional and pooling layers, followed by some consecutive fully connected layers, and, finally, a softmax layer to generate the desired outputs.

CNNs are trained with the back-propagation algorithm minimizing a cost function of the unknown weights. The cost function is an averaged value of the logarithmic representation of the probability by which each training image is correctly classified over the whole training set. It is represented as:

$$L=-\frac{1}{\left|X\right|}\sum_{i}^{\left|X\right|}ln\left(p\left(y^{i}|X^{i}\right)\right)$$

where $\left|X\right|$ denotes the number of training images, $X^{i}$ is the $i^{th}$ training image with the corresponding label $y^{i}$, and $p\left(y^{i}|X^{i}\right)$ is the probability value indicating the correct classification of $X^{i}$. A typical training algorithm is stochastic gradient descent for minimizing the cost function, the action of reducing the cost over the entire training set is approximated with the cost over mini-batches of data. The process of updating the weights follows the next formulations [49]:

$$\begin{array}{c} \gamma^{t}=\gamma^{\left\lfloor \frac{tN}{\left|X\right|}\right\rfloor }\\ V_{l}^{t+1}=\mu V_{l}^{t}-\gamma^{t}\alpha_{l}\frac{\partial \hat{L}}{\partial W_{l}}\\ W_{l}^{t+1}=W_{l}^{t}+V_{l}^{t+1} \end{array}$$

where $W_{l}^{t}$ denotes the weights in the *l*-th convolutional layer at iteration *t*, $\hat{L}$ means the cost over a mini-batch of size *N*, $\alpha_{l}$ is the learning rate of the *l*-th layer, μ is the momentum indicating the contribution of the previous weight update in the current iteration, and γ is the scheduling rate that decreases the learning rate α at the end of each epoch.
